# Supplementary material for: Estimated Glomerular Filtration Rate and Hearing Impairment in Japan: A Longitudinal Analysis Using Large-Scale Occupational Health Check-Up Data
Source: Int J Environ Res Public Health. 2022 Sep 28;19(19):12368. doi: 10.3390/ijerph191912368 (PMC9566123; doi:10.3390/ijerph191912368)
Supplement: Supplementary file 1 [file ijerph-19-12368-s001.zip › ijerph-1907349-supplementary.pdf]

Table S1. Associations of hearing impairment with all potential confounding factors in male participants.

		Low frequency (1000 Hz) HR (95% CI)[a]	High frequency (4000 Hz) HR (95% CI)[a]
Age (years), HR per 1 year increase		1.12 (1.10–1.13)	1.12 (1.11–1.12)
Hypertension	no	Ref.	Ref.
	yes[b]	1.05 (0.89–1.24)	0.98 (0.91–1.05)
Dyslipidemia	no	Ref.	Ref.
	yes[c]	1.01 (0.85–1.19)	0.93 (0.86–1.01)
Diabetes	no	Ref.	Ref.
	yes[d]	1.13 (0.89–1.42)	1.05 (0.94–1.17)
Body mass index (kg/m^2)			
<18.5		1 16 (0.81–1.67)	1.11 (0.95–1.31)
18.5–24.9		Ref.	Ref.
≥25.0		0.97 (0.81–1.16)	1.10 (1.02–1.19)
Smoking status			
Nonsmoker		Ref.	Ref.
Ex-smoker		1.04 (0.84–1.30)	1.19 (1.07–1.32)
≤20 cigarettes/day		1.21 (1.01–1.46)	1.57 (1.44–1.71)
>20 cigarettes/day		1.03 (0.78–1.35)	1.92 (1.72–2.15)
Alcohol consumption			
Nondrinker		Ref.	Ref.
Occasional drinker		1.08 (0.88–1.32)	0.91 (0.83–1.00)
Drinker <1 *go*/day[e]		1.06 (0.80–1.40)	0.97 (0.85–1.10)
Drinker ≥1 *go*/day[e]		1.09 (0.89–1.34)	1.02 (0.92–1.11)
Exercise	no	Ref.	Ref.
	yes[f]	0.98 (0.82–1.19)	1.01 (0.93–1.11)
Anemia	no	Ref.	Ref.
	yes[g]	1.06 (0.65–1.72)	1.28 (1.04–1.58)
Job type			
Office job		Ref.	Ref.
Professional job		1.25 (0.94–1.65)	2.17 (1.86–2.53)
Management		1.03 (0.76–1.41)	1.21 (1.01–1.43)
Sales		1.00 (0.68–1.48)	1.28 (1.04–1.57)
Service		1.24 (0.78–2.00)	1.72 (1.36–2.19)
Telecommunications		1.02 (0.69–1.50)	2.16 (1.81–2.59)
Manufacturing		1.51 (1.15–2.00)	2.94 (2.53–3.41)
Other		1.68 (1.19–2.37)	2.26 (1.88–2.71)

Notes: [a]Adjusted for estimated glomerular filtration rate and all other variables in this table. [b]Systolic blood pressure ≥130 mm Hg, diastolic blood pressure ≥85 mm Hg, and/or receiving medication. [c]Triglyceride level ≥150 mg/dL (1.7 mmol/L), high-density lipoprotein cholesterol level <40 mg/dL (1.03 mmol /L), and/or receiving medication. [d]Self-reported diagnosis of diabetes, receiving medication or HbA1c ≥6.5%. [e]One *go* contains approximately 23 g of ethanol. [f]Exercise causing light sweating ≥30 min/time (frequency, ≥2 days/week; duration, ≥1 year). [g]Hemoglobin (g/dL) ≤13 g/dL. Abbreviations: HR, hazard ratio; CI, confidence interval.

Table S2. Associations of hearing impairment with all potential confounding factors in female participants.

		Low frequency (1000 Hz) HR (95% CI)[a]	High frequency (4000 Hz) HR (95% CI)[a]
Age (years), HR per 1 year increase		1.10 (1.08–1.11)	1.14 (1.11–1.16)
Hypertension	no	Ref.	Ref.
	yes[b]	1.09 (0.88–1.35)	1.26 (0.99–1.60)
Dyslipidemia	no	Ref.	Ref.
	yes[c]	1.16 (0.91–1.48)	1.09 (0.83–1.43)
Diabetes	no	Ref.	Ref.
	yes[d]	1.37 (0.98–1.93)	1.47 (1.03–2.12)
Body mass index (kg/m^2)			
<18.5		1.36 (1.03–1.81)	0.91 (0.62–1.32)
18.5–24.9		Ref.	Ref.
≥25.0		1.14 (0.89–1.47)	1.18 (0.90–1.56)
Smoking status			
Nonsmoker		Ref.	Ref.
Ex-smoker		0.41 (0.22–0.76)	1.00 (0.62–1.63)
≤20 cigarettes/day		1.19 (0.92–1.54)	1.31 (0.98–1.75)
>20 cigarettes/day		1.25 (0.51–3.06)	1.03 (0.33–3.25)
Alcohol consumption			
Nondrinker		Ref.	Ref.
Occasional drinker		0.90 (0.72–1.12)	0.95 (0.74–1.23)
Drinker <1 *go*/day[e]		0.74 (0.47–1.15)	0.86 (0.53–1.40)
Drinker ≥1 *go*/day[e]		0.98 (0.66–1.44)	1.30 (0.87–1.93)
Exercise	no	Ref.	Ref.
	yes[f]	0.93 (0.69–1.24)	0.99 (0.73–1.37)
Anemia	no	Ref.	Ref.
	yes[g]	0.85 (0.64–1.14)	0.99 (0.72–1.39)
Job type			
Office job		Ref.	Ref.
Professional job		0.87 (0.52–1.44)	0.93 (0.51–1.70)
Management		1.26 (0.64–2.48)	1.04 (0.42–2.56)
Sales		1.87 (1.32–2.65)	1.52 (0.97–2.38)
Service		0.89 (0.55–1.44)	0.90 (0.52–1.57)
Telecommunications		– (No event)	– (No event)
Manufacturing		1.37 (1.07–1.74)	1.95 (1.48–2.57)
Other		1.24 (0.90–1.72)	1.35 (0.92–1.96)

Notes: [a]Adjusted for estimated glomerular filtration rate and all other variables in this table. [b]Systolic blood pressure ≥130 mm Hg, diastolic blood pressure ≥85 mm Hg, and/or receiving medication. [c]Triglyceride level ≥150 mg/dL (1.7 mmol /L), high-density lipoprotein cholesterol level <50 mg/dL (1.3 mmol/L), and/or receiving medication. [d]Self-reported diagnosis of diabetes, receiving medication, or HbA1c ≥6.5%. [e]One *go* contains approximately 23 g of ethanol. [f]Exercise causing light sweating ≥30 min/time (frequency, ≥2 days/week; duration, ≥1 year). [g]Hemoglobin (g/dL) ≤12 g/dL. Abbreviations: HR, hazard ratio; CI, confidence interval.
